# Supplementary material for: Quantitative Histopathology of Stained Tissues using Color Spatial Light Interference Microscopy (cSLIM)
Source: Sci Rep. 2019 Oct 11;9:14679. doi: 10.1038/s41598-019-50143-x (PMC6789107; doi:10.1038/s41598-019-50143-x)
Supplement: Supplementary file 1 — Quantitative Histopathology of Stained Tissues using Color Spatial Light Interference Microscopy (cSLIM): Supplementary Information [file 41598_2019_50143_MOESM1_ESM.pdf]

Quantitative Histopathology of Stained Tissues using Color Spatial Light Interference Microscopy (cSLIM): Supplementary Information

Hassaan Majeed[1], Adib Keikhosravi[2], Mikhail E. Kandel[3], Tan H. Nguyen[4], Yuming Liu[2], Andre Kajdacsy-Balla[5], Krishnarao Tangella[6], Kevin W. Eliceiri[2], Gabriel Popescu[1,3].*

[1] Quantitative Light Imaging (QLI) Lab, Department of Bioengineering, Beckman Institute of Advanced Science and Technology, University of Illinois at Urbana Champaign, 405 N. Matthews, Urbana, IL 61801, USA.

[2] Laboratory for Optical and Computational Instrumentation (LOCI), Department of Biomedical Engineering, University of Wisconsin-Madison, Madison, WI 53706, USA.

[3] Quantitative Light Imaging (QLI) Lab, Department of Electrical and Computer Engineering, Beckman Institute of Advanced Science and Technology, University of Illinois at Urbana Champaign, 405 N. Matthews, Urbana, IL 61801, USA.

[4] Biomedical Optics and Biophotonics group, Department of Electrical Engineering and Computer Science, Massachusetts Institute of Technology, Cambridge, MA 02142, USA.

[5] Department of Pathology, University of Illinois at Chicago, 840 South Wood Street, Suite 130 CSN, Chicago, IL 60612, USA.

[6] Christie Clinic, 1400 West Park Street, Urbana, IL 61801, USA.

*Direct all correspondences to: Email: gpopescu@illinois.edu; Tel: +1(217) 333-4840; Fax: +1(217) 244-1995

S.1 SLM calibration with RGB camera

SLIM requires SLM calibration so that the correct phase modulation is applied between the scattered and incident components of light [1]. As shown in Figs. 1 (e) and (f) in the main text, the spectrum of incident light is different from the equivalent cSLIM spectrum in both spectral width and central wavelength. For these reasons, in contrast with grayscale imaging, recalibration of the SLM was required for the cSLIM system. After configuring the SLM in amplitude modulation mode [1], the 8-bit grayscale input to the SLM was scanned from 0-255. The corresponding amplitude modulation in $I(x, y; \varphi)$ [given by Eq. (1) in the main text] was measured and each frame was averaged to generate a one dimensional amplitude modulation curve, shown in Fig. S1 (a). By taking the Hilbert transform of this curve, the SLM calibration curve [Fig. S1 (b)], relating phase values to grayscale input, was obtained. The grayscale values corresponding to $\varphi = 0, \pi/2, \pi, 3\pi/2$ rad were used in all imaging experiments.

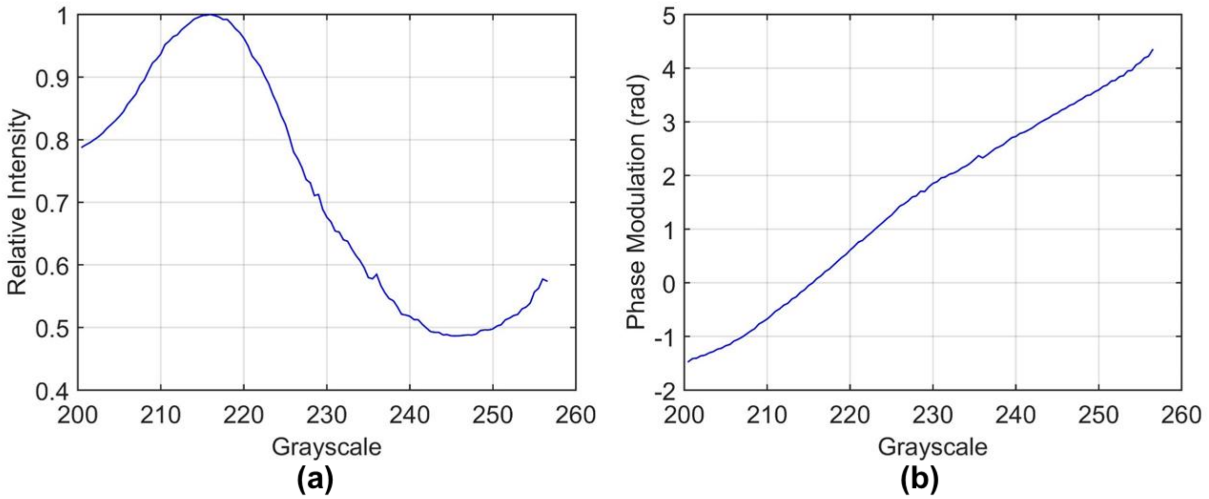

Figure S1. (a) Amplitude (intensity) modulation curve obtained in cSLIM by configuring the SLM in amplitude mode. **(b)** Phase calibration extracted using the Hilbert transform of the curve from (a).

S.2 Attenuation calibration for the bright-field objective

Phase contrast objectives contain a phase plate in their back focal planes that not only introduces a $\pi/2$ rad phase shift between the unscattered light U_0 and scattered light U_1 but also imparts an attenuation factor $\alpha_{pc} = \frac{|U_1|}{|U_0|}$. This factor is used during SLIM phase reconstruction while calculating the ratio of the amplitudes of the two interfering fields [1,2]. In our cSLIM experiments, we have used a bright-field objective that does not have this attenuating element. An equivalent value of attenuation, α_{bf} , was, therefore, used during phase reconstruction. α_{bf} was obtained through the following calibration procedure, which is illustrated in Fig. S2. An unstained TMA core was imaged using cSLIM with both a phase contrast and a bright-field objective. During phase reconstruction for the phase contrast case, the measured attenuation factor $\alpha_{pc} = 1.97$ was used and the raw phase image $\phi(x, y)$ was obtained [Fig. S2 (c)]. For the bright-field case, the equivalent attenuation α_{bf} was numerically tuned and the phase $\phi(x, y)$ was obtained for each α_{bf} . For each α_{bf} , we calculated the cross-correlation $\gamma(P_{pc}, P_{bf})$ between the probability distributions P_{pc} and P_{bf} of $\phi(x, y)$ in the phase contrast and bright-field cases, respectively [3]. P_{pc} and P_{bf} were obtained by normalizing their respective image histograms (constructed with 512 bins each). As shown in Fig. S2 (b), $\gamma(P_{pc}, P_{bf})$ maximizes at $\alpha_{bf} = 3.4$, which was, thus, the value used for all subsequent imaging experiments. Fig. S2 (d) shows the phase image obtained using the bright-field objective at $\alpha_{bf} = 3.4$. It has similar phase values compared to those obtained using a phase contrast objective [Fig. S2 (c)].

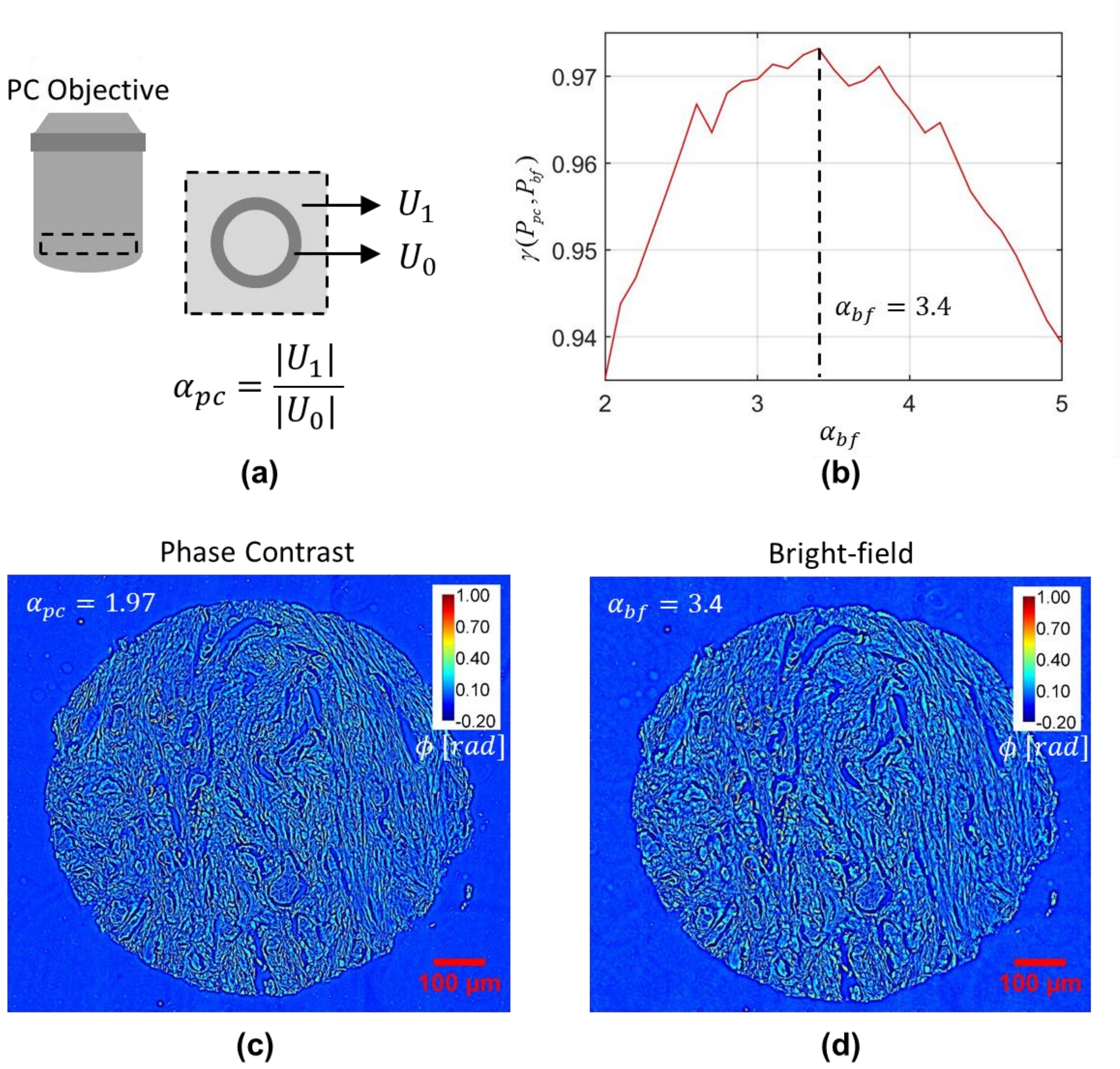

Figure S2. (a) Attenuation α_{pc} of the unscattered light U_0 with respect to scattered light U_1 in a phase contrast objective. For imaging with a bright-field objective this term is introduced numerically, α_{bf}. **(b)** Cross-correlation coefficient γ between the probability distributions P_{pc} and P_{bf} of the same unstained TMA core measured using phase contrast and bright-field objectives, respectively. γ is measured as a function of α_{bf} and peaks at α_{bf} = 3.4. **(c)** cSLIM image of the unstained tissue core obtained using a phase contrast objective with the measured value of attenuation $\alpha_{pc} = 1.97$. **(d)** cSLIM image of the unstained tissue core obtained at the optimum value of α_{bf} = 3.4.

S.3 Procedure for stain normalization

As discussed in Results and Discussion of the main text, we extracted the normalized phase maps $Z(x, y)$ from the raw phase maps $\phi(x, y)$ generated by the cSLIM system, using Eq. (4).

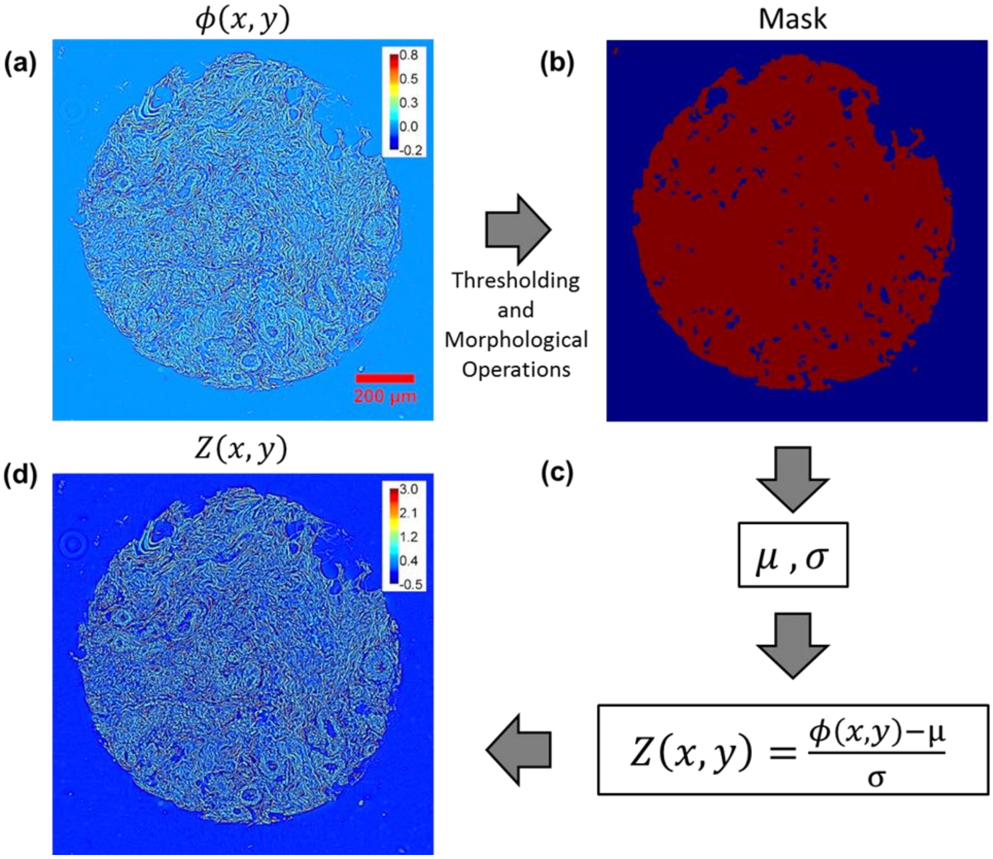

Figure S3. (a) Raw phase map of stained tissue microarray core. **(b)** Mask for computation of μ and σ over foreground pixels. **(c)-(d)** Computation of normalized phase map.

This computation is illustrated in Fig. S3. For each tissue core $\phi(x, y)$ map [Fig. S3 (a)], we first generated a segmentation mask [Fig. S3 (b)] using thresholding followed by morphological operations (closing and removal of all 8-neighboring connected objects with total area smaller than 1600 μm^2). A circular structural element was used for morphological closing with a diameter of approx. 4 μm . The mask was then used to calculate the mean μ and standard deviation σ of the

foreground region in $\phi(x,y)$ (region occupied by tissue core). Finally, the $Z(x,y)$ image was computed for the core from these parameters as shown in Figs. S3 (c) and (d).

S.4 Analysis of dispersion in stained tissue

As discussed in the main text, the incident light and cSLIM spectra are different due to the effect of the spectral responses of the red, green, and blue channels of the cSLIM system. This results in different values of both the central wavelength and the coherence length for RGB and grayscale imaging even for an unstained tissue sample [see Figs. 1 (e) and (f) in the main text]. In the presence of H&E stained tissue, these two spectral parameters are expected to change even further due to absorption in dye. Our results suggest that normalization of both stained and unstained tissue phase maps [using Eq. (4) in the main text] accounts for these differences, making results from both cases very similar. We detail in this section reasons for why this normalization removes the stain dependent signal from phase images.

To explore this, we first compute the pixel-wise spectra of light detected by the RGB camera when an H&E stained tissue core is imaged. From the bright-field image measured by the camera [illustrated in Fig. S4 (a)], we extract the red, green and blue channel intensity images, $R(x,y), G(x,y)$, and $B(x,y),$ respectively. By dividing each image by the average signal in a 30 x 30 pixel background region within it (where no tissue is present), we are able to obtain the transmission images for three channels: $t_R(x,y), t_G(x,y)$ and $t_B(x,y)$ [Fig. S4 (b)]. Fig. S4 (c) shows the spectral responses, in the absence of tissue absorbance, of the red, green and blue channels [$S_R(\lambda), S_G(\lambda)$ and $S_B(\lambda)$, respectively]. These responses include both the filter responses of the camera as well as the weights attached to the three channels numerically during computation

of the equivalent grayscale image $I(x, y)$ [Eq. (1) in the main text]. Thus, in the *absence* of tissue absorbance, the spectrum for cSLIM imaging, $S_c(\lambda)$, can be computed as

$$S_c(\lambda) = S_i(\lambda)[S_R(\lambda) + S_G(\lambda) + S_B(\lambda)], \quad \text{(S1)}$$

where $S_i(\lambda)$ is the incident light spectrum. $S_i(\lambda)$ and $S_c(\lambda)$ are plotted in Fig. 1(e) of the main text.

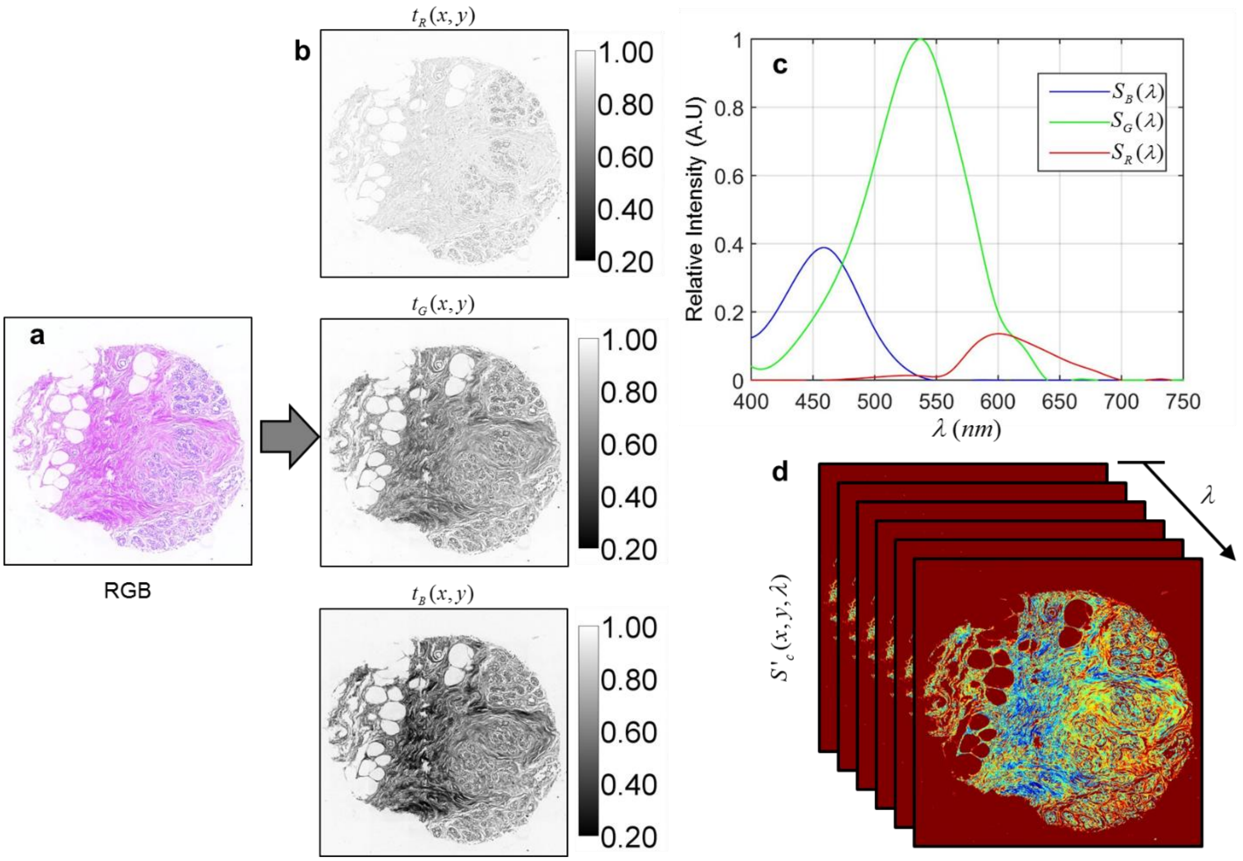

Figure. S4 (a) Bright-field image of a stained TMA core. **(a)** Transmission maps for the red, green and blue channels. **(c)** Spectral response of cSLIM for the red, green and blue channels in the absence of tissue absorbance. **(d)** cSLIM spectrum at each pixel computed using Eq. (S2).

Assuming constant values of transmission for each spectral channel per pixel [$t_R(x, y)$, $t_G(x, y)$, and $t_B(x, y)$] the spectral response for each channel in the *presence* of tissue absorbance

can be calculated as $S'_j(x,y,\lambda) = S_j(\lambda) t_j(x,y)$ where $j = R, G, B$. Multiplying each response by the illumination spectrum $S_i(\lambda)$ and summing them gives us the pixel-wise cSLIM spectrum in the presence of tissue absorbance, namely

$$S'_c(x,y,\lambda) = S_i(\lambda)[S'_R(x,y,\lambda) + S'_G(x,y,\lambda) + S'_B(x,y,\lambda)]. \tag{S2}$$

This spatially-resolved spectrum is illustrated in Fig. S4 (d). From $S'_c(x,y,\lambda)$ we can compute the central wavelength map $\lambda'_c(x,y)$ for the stained tissue core. By subtracting the mean value of λ'_c in a 30 x 30 pixel background region from $\lambda'_c(x,y)$, we obtain the map of the wavelength shift due to tissue absorbance, $\delta\lambda'_c(x,y)$, illustrated in Fig. S5 (a). This image shows that dispersion (variation of λ'_c in x and y) due to tissue absorbance is small. To quantify this effect, we calculated the histogram (computed over the foreground region consisting of tissue only) in Fig. S5 (b) which shows a mean shift of 2.3 nm with a standard deviation of 1.5 nm. Absorbance related spectral changes also cause modifications in the coherence length l'_c across the stained tissue. The image $l'_c(x,y)$ can be obtained from $S'_c(x,y,\lambda)$ by computing its autocorrelation function through Fourier transformation and measuring the full-width half maximum (FWHM) of the function's envelope [see Results and Discussion in the main text]. The shift in coherence length $\delta l'_c(x,y)$ can be extracted from $l'_c(x,y)$ by subtracting from it the mean value of l'_c in a 30 x 30 pixel background region. $\delta l'_c(x,y)$ is shown in Fig. S5 (c) and its histogram is shown in Fig. S5 (d). We measured a mean of $-81.0\,nm$ and a standard deviation of $58.3\,nm$ for $\delta l'_c(x,y)$.

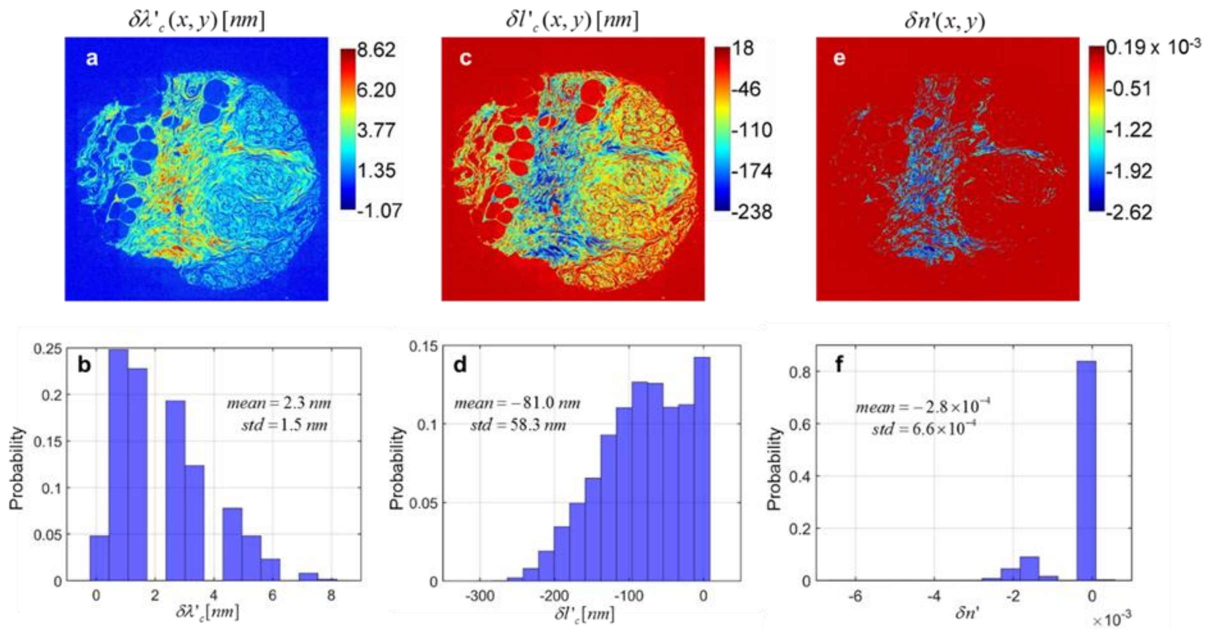

Figure S5. (a) Central wavelength shift across the tissue core due to dispersion. **(b)** Normalized histogram of the image in (a) over the foreground (tissue region). **(c)** Shift in coherence length across tissue core due to dispersion. **(d)** Normalized histogram of the image in (c) over the foreground (tissue region). **(e)** Dispersion in the stained tissue core. **(f)** Normalized histogram of the image in (e) over the foreground (tissue region).

This spectral analysis sheds light on why a simple division by the standard deviation of $\phi(x, y)$ results in the stain-independent map $Z(x, y)$ [Eq. (4) in main text]. The slight difference in central wavelength between incident light and cSLIM (589 nm versus 558 nm) would indicate that cSLIM should have different phase values from gray scale imaging even for *unstained* tissue. However, division of $\phi(x, y)$ by its standard deviation removes the central wavelength dependence of $Z(x, y)$

$$Z(x, y) = \frac{n(x, y) - mean\{n(x, y)\}}{std\{n(x, y)\}}. \tag{S3}$$

Here it is assumed that the *unstained* tissue refractive index $n(x, y)$ is independent of wavelength as well (a reasonable assumption given the small difference in central wavelength between incident

light and cSLIM spectra). The operators $mean\{.\}$ and $std\{.\}$ refer to the spatial mean and spatial standard deviation, respectively.

For *stained* tissue, the central wavelength λ'_c itself is x and y dependent and does not cancel out when $\phi(x,y)$ is divided by its standard deviation. From Eq. (4) in the main text, the normalized phase image for stained tissue Z', measured by cSLIM, is thus given by

$$Z'\left[x,y,\lambda'_c(x,y)\right]=\frac{\dfrac{\Delta n'\left[x,y,\lambda'_c(x,y)\right]}{\lambda'_c(x,y)}-mean\left\{\dfrac{\Delta n'\left[x,y,\lambda'_c(x,y)\right]}{\lambda'_c(x,y)}\right\}}{std\left\{\dfrac{\Delta n'\left[x,y,\lambda'_c(x,y)\right]}{\lambda'_c(x,y)}\right\}} \quad \text{(S4a)}$$

$$Z'[x,y,\lambda'_c(x,y)]=\frac{\dfrac{\Delta n'(x,y,\lambda_c)+\delta n'[x,y,\delta\lambda'_c(x,y)]}{\lambda_c+\delta\lambda'_c(x,y)}-mean\left\{\dfrac{\Delta n'(x,y,\lambda_c)+\delta n'[x,y,\delta\lambda'_c(x,y)]}{\lambda_c+\delta\lambda'_c(x,y)}\right\}}{std\left\{\dfrac{\Delta n'(x,y,\lambda_c)+\delta n'[x,y,\delta\lambda'_c(x,y)]}{\lambda_c+\delta\lambda'_c(x,y)}\right\}}$$

(S4b)

where λ_c is the cSLIM central wavelength (558 nm) in the absence of tissue absorbance and $\delta\lambda'_c$ is the shift in central wavelength caused by staining such that $\lambda'_c(x,y)=\lambda_c+\delta\lambda'_c(x,y)$. $\Delta n'[x,y,\lambda'_c(x,y)]=n'[x,y,\lambda'_c(x,y)]-n_0$ with n_0 the refractive index of the medium surrounding the tissue and n' the refractive index of *stained* tissue. $\delta n'$ is the shift in stained tissue refractive index due to stain induced dispersion i.e $n'[x,y,\lambda'_c(x,y)]=n'(x,y,\lambda_c)+\delta n'[x,y,\delta\lambda'_c(x,y)]$.

Equations (S4a) and (S4b) would indicate the need for a local normalization constant rather than the global one we have used in our study, in order to make the results from stained and

unstained tissue comparable. However, if $\delta\lambda'_c$ and $\delta n'$ are small valued and/or have weak x and y dependence (small spatial variance), Eq. (S4b) becomes approximately

$$Z'(x,y,\lambda_c) = \frac{n'(x,y,\lambda_c) - mean\{n'(x,y,\lambda_c)\}}{std\{n'(x,y,\lambda_c)\}}. \tag{S5}$$

Due to weak dispersion, $n'(x,y,\lambda_c) \approx n(x,y)$, meaning Eq. (S5) is approximately equal to Eq. (S3) and $Z' \approx Z$. As shown in Fig. S5, the x and y dependence of $\delta\lambda'_c$ is indeed small (standard deviation of 1.5 nm over the image). To determine this for $\delta n'$, the following procedure was employed. The 3D spectrum $S'_c(x,y,\lambda)$, was first rescaled and resampled to obtain the frequency spectrum $S'_c(x,y,\omega)$ where $\omega = 2\pi c/\lambda$, c being the speed of light in air. The same was done to $S_c(\lambda)$ to obtain $S_c(\omega)$. The transmission spectrum $T(x,y,\omega)$ was then obtained as

$$T(x,y,\omega) = \frac{S'_c(x,y,\omega)}{S_c(\omega)}. \tag{S6}$$

From the transmission spectrum, the refractive index of stained tissue $n'(x,y,\lambda)$ can be obtained by using the Hilbert transform relationship between the real and imaginary parts of the electric susceptibility [4,5]. The procedure for this is outlined in Ref. [4]. The resulting refractive index is determined only up to an additive constant since the Hilbert transform of a constant is zero. Since we have knowledge of the central wavelength at each pixel [given by $\lambda'_c(x,y)$], the refractive index map $n'[x,y,\lambda_c(x,y)]$ at this central wavelength can be computed from $n'(x,y,\lambda)$. Finally, by subtracting the refractive index map in absence of dispersion, $n'(x,y,\lambda_c)$, from $n'[x,y,\lambda_c(x,y)]$ we get the shift in refractive index due to tissue absorption $\delta n'[x,y,\lambda_c(x,y)]$ or simply $\delta n'(x,y)$. This subtraction also accounts for the differences in additive constants across the $n'[x,y,\lambda_c(x,y)]$ map

caused by the computation of an independent Hilbert transform per pixel. $\delta n'(x, y)$ is shown in Fig. S5 (e) whereas Fig. S5 (f) shows its histogram, once again computed only over the foreground (tissue region). As shown, not only is there a small shift in refractive index due to dispersion in tissue but the x and y dependence is also weak (standard deviation of 6.6 x 10^{-4}).

Thus, we conclude that our normalization works well despite tissue absorbance because of the small change in λ'_c, and thus in refractive index n', across the tissue core. Any constant (spatially invariant) changes in both wavelength and refractive index are accounted for in $Z(x, y)$ by subtraction by the mean and division by the standard deviation of the raw phase $\phi(x, y)$.

References

1 Wang, Z. *et al.* Spatial light interference microscopy (SLIM). *Optics Express* **19**, 1016-1026, doi:10.1364/OE.19.001016 (2011).

2 Popescu, G. *et al.* Fourier phase microscopy for investigation of biological structures and dynamics. *Optics Letters* **29**, 2503-2505, doi:10.1364/OL.29.002503 (2004).

3 Bracewell, R. *The Fourier Transform and Its Applications*. (McGraw-Hill, 1965).

4 Whittaker, K. A., Keaveney, J., Hughes, I. G. & Adams, C. S. Hilbert transform: Applications to atomic spectra. *Physical Review A* **91**, 032513 (2015).

5 Lucas, J., Géron, E., Ditchi, T. & Holé, S. A fast Fourier transform implementation of the Kramers-Kronig relations: Application to anomalous and left handed propagation. *AIP Advances* **2**, 032144, doi:10.1063/1.4747813 (2012).
